# The effect of oral contraceptive pills on the macula, the retinal nerve fiber layer, the ganglion cell layer and the choroidal thickness

**DOI:** 10.1186/s12886-019-1263-2

**Published:** 2019-12-10

**Authors:** Yasmine Maher Shaaban, Tamer Abdel Fattah Badran

**Affiliations:** 1 Department of Ophthalmology, Faculty of Medicine, Ain Shams University, Abbassyia, Cairo, 11566 Egypt; 2 The Eye Subspecialty Center, (ESC), 18 El Khalifa El Maamoun Street, Heliopolis, Cairo, 11402 Egypt; 3 Cairo, Egypt

**Keywords:** Oral contraceptive, Macula, Nerve fiber, Ganglion, Choroid, Ocular coherence tomography

## Abstract

**Backgroun:**

To evaluate the effect of oral contraceptive pills (OCP) on the macula, the retinal nerve fiber layer (RNFL), the ganglion cell layer (GCL), and the choroidal thickness (CT).

**Methods:**

In this prospective observational cross-sectional study, 60 eyes of 30 healthy women taking monophasic OCP (0.03 mg ethinylestradiol and 0.15 mg levonorgestrel) for contraception for at least 1 year were compared with 60 eyes of a control group of 30 healthy women who were not taking any OCP. Spectral-Domain Optical Coherence Tomography (SD-OCT) was used to evaluate the macula, the RNFL, the GCL, and the CT. Measurements were taken in the follicular phase (day 3) of the last menstrual cycle in all women. The body mass index (BMI) scores of all participants were also recorded.

**Results:**

No disparity in terms of age and BMI between both groups was observed (*p* = 0.444, *p* = 0.074, respectively)*.* All the macular parameters measurements were considerably lower in the OCP group compared to the control group (*p* < 0.001). Also, the RNFL thickness, the GCL thickness, and the CT were all significantly thinner in the OCP group (*p* < 0.001).

**Conclusions:**

The use of OCP can cause significant changes in the retina and choroid thickness over 1 year period. The women who are using OCP for a longer duration could have some eye problems. OCT should be routinely done for follow up. Further long term studies are required, using different preparations of OCP. It is important to find out when this thickness alterations can be clinically significant or symptomatic and if these changes are reversible or not.

## Background

Oral contraceptive pills (OCP) are commonly used by women all over the world during their reproductive life. Several studies reported the occurrence of a variety of ocular disorders in women using OCP [1]. Estrogen and progesterone receptors have been observed in multiple ocular tissues, such as the choroid, retina, lens, conjunctiva, cornea, and the Meibomian gland [2]. Sex hormones proved to affect the retina and the choroid and a correlation between sex hormones and retinal disorders have been described [2–4]. The macula is the central part of the retina and any degenerative changes in this area could affect the central vision. The macula, the (RNFL), the ganglion cell layer (GCL), and the choroidal thickness (CT) may decrease with age, degenerative diseases, ischemia, chronic inflammatory processes [5]. These changes may also be due to genetic, environmental, and hormonal factors [6]. Since oral contraceptive pills (OCP) usually contain estrogen and progesterone, the retina and choroid are at risk. In literature, there is very little data concerning the effect of OCP on the thickness of the macula, the RNFL, the GCL, and the CT [7]. In this study, we aim to evaluate the effect of OCP on the macula, the NFL, GCL, and the CT, to address any reported complications or side effects to people who use OCP for contraception or for other reasons other than contraception and to consider regular ocular examination and follow up while using these pills.

## Methods

### Aim

Is to evaluate the thickness of the macula, the RNFL, the GCL, and the choroid using SD-OCT in women using the same OCP and compare them with those who do not use any OCP. Sixty eyes of 30 healthy women who had received OCP for more than 1 year (the OCP group) were compared with 60 eyes of healthy women who were not using any OCP (the control group). All participants were Egyptian women, in their reproductive age period of 23–36 years. Those who do not use OCP have a regular natural menstrual cycle of 28–30 days. All participants were evaluated in the follicular phase (day3) of their last menstrual cycle.

### Design

An observational comparative cross-sectional study.

### Setting

The study was performed in The Eye Subspecialty Center (ESC), Heliopolis, Cairo, Egypt.

The cases were recruited from patients visiting the Outpatient Clinic, and the OCT was performed in the Investigation Unit. All procedures in this study adhered to the Declaration of Helsinki’s ethical principles for medical research involving human subjects and were approved by the Ethics Committee Of The Eye Subspecialty Center (ESC), 18 El Khalifa El Maamoun Street, Heliopolis, 11,402 Cairo, Egypt. Verbal informed consent was obtained from all participants.

All candidates had a complete ophthalmologic examination including medical history, visual acuity, cycloplegic refraction, Goldmann applanation tonometry, slit-lamp bio microscopy, and fundus examination. Those with the best-corrected visual acuity of ≥20/25, spherical refraction within ±3.00 D, and cylinder refraction within ±1.50 D, and had normal ocular examination results were included in the study. Body mass index (BMI) of all candidates was also measured and compared in both groups based on the formula: BMI = (weight/height^2^ [kg/m^2^]).

#### Exclusion criteria

Those who had previous ocular surgery, ocular trauma, uveitis, glaucoma, cataract, macular degeneration, and cystoid macular edema were excluded from the study. Pregnancy, diabetes mellitus, hypertension, cardiovascular events, thyroid disorders, Cushing disease, thromboembolic disease, cancer, chronic liver disease, kidney disease, pancreatitis, congenital adrenal hyperplasia, psychotic disorders, and those who use antidepressants, steroidal hormone, mood stabilizers, caffeine or tobacco were also excluded from the study.

The used OCP is a commonly prescribed type of combined oral contraceptive (COC) birth control in Egypt. It is monophasic pills having estrogen (0.03 mg ethinylestradiol) and progestin (0.15 mg levonorgestrel). Monophasic pills contain equal amounts of the hormones estrogen and progestin for an entire monthly cycle.

Spectral-Domain Optical Coherence Tomography (SD-OCT), Cirrus HD-OCT model 5000 (Carl Zeiss Meditec, Germany) was used to measure the thickness of macula, the RNFL, the GCL, and the CT. The procedure was done by the same person using the same OCT unit. The Scan Protocol was done as follows: Macular Cube Scan 512 × 128 was used for macular thickness, cube volume, cube average thickness, and ganglion cell layer thickness. The Optic Disc Cube 200 × 200 SCAN was used for RNFL thickness analysis. The Enhanced Depth Image Scan was used for choroidal thickness, in which three points were measured, subfoveal, 3 mm point nasal to the fovea and 3 mm point temporal to the fovea. The study was done in the period between January 2017 and October 2018.

### Statistics

Quantitative data were presented as mean and standard deviation. Independent samples t-test was used to test the statistical significance of the difference of means of the two groups. *P* values ≤0.05 were considered significant. The Statistical Package for the Social Sciences SPSS version 20 for Windows (IBM Corp., Armonk, NY) was used for the statistical analysis.

## Results

There was no disparity in terms of age and BMI between both the studied groups. The OCP group had a mean age of 30.48 ± 3.4 years while the control group had a mean age of 29.97 ± 3.87 years (t = 0.777, *p* = 0.444). The mean BMI scores was 23.3 + 2.2 kg/m^2^ for the OCP group and 22.3 + 2.3 kg/m^2^ for the control group (t = 1.818, *P* = 0.074). BMI of 18.5–24.9 kg/m^2^ is considered as normal**.** The difference between the two groups was statistically insignificant (Table 1). The OCP used in this study had a significant thinning effect on all the parameters of all parts of the macula (*p* < 0.001), all parts of RNFL (*p* < 0.001), all parts of GCL (*p* < 0.001), subfoveal choroid, temporal and nasal choroid 3 mm points nasal and temporal to the fovea (*p* < 0.001) (Tables 2, 3, 4).

**Table 1:** Demographic data for oral contraceptive pills group and the control group

Parameter	OCP Group (*n* = 60)	Control Group (*n* = 60)	t	p
Age, years	30.48 ± 3.4	29.97 ± 3.87	0.777	0.444
Gender	female	female		
Number of subjects	30	30		
Ethnicity	Egyptians	Egyptians		
Study location	Cairo, Egypt	Cairo, Egypt		
BMI. kg/m^2^	23.3 + 2.2	22.3 + 2.3	1.818	0.074

*OCP* Oral contraceptive pills, *BMI* Body Mass Index, Values are expressed as mean ± SD. *P* values ≤0.05 were considered significant

**Table 2:** Comparison of macular and choroidal thickness in women using oral contraceptive pills and the control group

Parameter (μm)	OCP group (*n* = 60)	Control group (*n* = 60)	t	*p*-value
	Mean ± SD	Mean ± SD		
MACULA				
Central subfield	242.75 ± 10.22	268.13 ± 11.86	12.556	< 0.001
Superior inner macula	293.88 ± 10.48	308.95 ± 10.94	7.704	< 0.001
Temporal inner macula	295.48 ± 7.58	307.85 ± 9.61	7.828	< 0.001
Inferior inner macula	295.63 ± 8.52	310.57 ± 11.06	8.288	< 0.001
Nasal inner macula	298.95 ± 10.31	314.57 ± 7.89	9.316	< 0.001
Average inner macula	296.0 ± 8.05	310.48 ± 7.94	9.922	< 0.001
Superior outer macula	269.13 ± 8.49	282.97 ± 11.31	7.577	< 0.001
Temporal outer macula	271.68 ± 15.43	284.45 ± 11.59	5.124	< 0.001
Inferior outer macula	268.05 ± 7.44	286.38 ± 10.40	11.108	< 0.001
Nasal outer macula	271.93 ± 9.15	301.22 ± 13.33	14.031	< 0.001
Average outer macula	270.2 ± 8.27	288.75 ± 8.71	11.962	< 0.001
Cube volume s (mm^3^)	8.71 ± 0.50	10.05 ± 0.38	16.539	< 0.001
Cube average thickness	273.52 ± 5.51	284.73 ± 6.18	10.496	< 0.001
CHOROID				
Temporal choroid	219.87 ± 6.26	259.82 ± 14.50	19.595	< 0.001
sub foveal choroid	233.98 ± 9.34	272.38 ± 15.50	16.433	< 0.001
Nasal choroid	223.12 ± 7.12	260.23 ± 16.82	15.738	< 0.001
Average choroid	225.66 ± 7.12	264.14 ± 14.75	18.2	< 0.001

*OCP* Oral contraceptive pills. Values are expressed as mean ± SD. *P* values ≤0.05 were considered significant

**Table 3:** Average peripapillary retinal nerve fiber layer thickness in women using oral contraceptive pills and the control group

Parameter (μm)	OCP group (*n* = 60)	Control group (*n* = 60)	t	*p*-value
	Mean ± SD	Mean ± SD		
superior	103.38 ± 9.97	121.22 ± 7.20	11.232	< 0.001
Temporal	59.83 ± 7.22	75.60 ± 11.60	8.937	< 0.001
inferior	112.28 ± 14.80	133.72 ± 7.01	10.137	< 0.001
Nasal	73.05 ± 9.93	99.67 ± 11.42	13.624	< 0.001
Average	87.14 ± 8.77	107.55 ± 5.00	15.661	< 0.001

*OCP* Oral contraceptive pills. Values are expressed as mean ± SD. *P* values ≤0.05 were considered significant

**Table 4:** Average Ganglion cell layer thickness in women using oral contraceptive pills and the control group

Parameter (μm)	OCP group (*n* = 60)	Control group (*n* = 60)	t	*p*-value
	Mean ± SD	Mean ± SD		
superior	76.07 ± 7.59	86.78 ± 4.88	9.201	< 0.001
Superior Temporal	76.07 ± 6.97	87.05 ± 4.98	9.928	< 0.001
Inferior Temporal	76.38 ± 6.69	87.93 ± 4.07	11.428	< 0.001
Inferior	75.80 ± 6.67	87.38 ± 4.20	11.386	< 0.001
Inferior Nasal	76.35 ± 7.30	88.05 ± 3.84	10.987	< 0.001
Superior Nasal	76.55 ± 7.00	87.55 ± 3.60	10.830	< 0.001
Average	76.20 ± 6.68	87.46 ± 3.40	11.631	< 0.001

*OCP* Oral contraceptive pills. Values are expressed as mean ± SD. *P* values ≤0.05 were considered significant

## Discussion

Estrogen and progesterone receptors have been observed in different ocular tissues, such as the cornea, the conjunctiva, the Meibomian glands, the lens, the choroid, and the retina. The expression of estrogen and progesterone receptors in the eye is responsible for their ocular effects and various studies published in the literature about these receptors and their role in changing eye structures [2]. Oral Contraceptive Pills are widely used for contraception, but they are also indicated in menorrhagia, endometriosis, acne and hirsutism, fibroid uterus and premenstrual syndrome [8].

Reviewing the impact of combined oral contraceptives on ocular tissues showed that the OCP has multiple adverse effects on ocular tissues [1], which include dry eye symptoms related to decreased lipid production [9], asymptomatic corneal edema, contact lenses discomfort and intolerance [10]. Significant increase in the central corneal thickness values was also reported in patients using OCP [11]**.** There are also retinal neuro-ophthalmologic complications involving the 6th cranial nerve paralysis, parietal syndrome, hemianopsia, papillary edema, and retrobulbar neuritis [12]. The vascular complications of OCP include central retinal artery or vein occlusion, intraocular hemorrhages, aneurysms, macular or disc edema, and acute ischemic optic neuropathy [13]. Also, they have been associated with high rates of cardiovascular events, venous thromboembolic disease, ischemic strokes, and breast cancer [14, 15].

There are also some studies concerning the effect of sex hormone fluctuations during the menstrual cycle on the eye. Some researchers have shown that these hormone fluctuations exhibited correlations with changes in ocular tissue. Tear production, stability, dryness, and inflammation were significantly related to hormonal fluctuations in the menstrual cycle. Impairment of these functions appeared to be related to the estrogen peak during the follicular phase, especially in patients with a dry eye [16]. During the menstrual cycle, the corneal thickness is thinner at the beginning of the cycle and thicker at the end [17]. Evaluation of the effects of the menstrual cycle on the choroidal thickness of healthy women of reproductive age showed a significant decrease in thickness in the mid-luteal phase of the menstrual cycle [18]. Also, the choroidal thickness measured by SD-OCT was reported to be significantly thinner in postmenopausal women than healthy reproductive-age women, which speculated to be secondary to menopausal estrogen deficiency [19].

In this study, we were evaluating the thickness of the macula, the RNFL, GCL, and CT in women who received OCP (0.03 mg ethinylestradiol and 0.15 mg levonorgestrel) regularly for at least 1 year. We observed that the thickness of all the macular parameters, the cube macular volume, the RNFL, the GCL, and the CT decreased significantly in the OCP group compared to the control group. These thinning effect of the OCP are similar to age-related hormonal changes in which thinning of the macula is due to macular atrophy or RNFL atrophy [20]. In this study, these findings were not considered related to age since the average age of the women was 30.48 ± 3.4 years.

Apart from the study done by Madendag et al. [7] in 2017, concerning the effect of OCP on the thickness of the macula, the RNFL and the choroid using OCT, there are no other available data. Madendag et al. used OCP containing 0.03 mg ethinylestradiol and 3 mg drospirenone. They found that all OCT measurements of macular parameters were considerably decreased in the OCP group compared to the control group except the foveal center thickness which remained unchanged. They speculated that the absence of blood vessels in the fovea could be the cause of unaffected fovea. They also found that only the average of the RNFL, the nasal-inferior, and the temporal-inferior parts of the RNFL was considerably slimmer in the OCP group but the other parts of the RNFL remained unchanged. They also reported unchanged CT. They speculated that using a combined oral contraceptive could be the cause of unaffected CT since the estrogen is opposed by the progesterone.

In accordance with our study is that all macular variable values were considerably lower in the OCP group compared to the control. The same was applied to the cube macular volume which decreased in both studies. Controversial with our results is reporting thinning of only parts of the peripapillary RNFL and not all the parts. They also reported unaffected choroid. We reported significant thinning of all parts of RNFL, all parts, of GCL and the choroid.

We also compared our data of the control group regarding the MT, CT, and NFL thickness (Tables 2 and 3) with the normative data of other studies using SD-OCT. Liu et al. [21], using Cirrus HD-OCT reported CST of 262.4 ± 22.8 μm, macular thickness (MT) of 281.3 ± 14.5 μm, and macular volume (MV) of 10.1 ± 0.6 mm^3^, respectively. Manjunath et al. [22], reported mean subfoveal (CT) of 272 ± 81 μm, showing a thin choroid nasally, thickest choroid sub-foveally, and thinner choroid temporally. Using Spectralis SD-OCT, Bindu et al. [23], provided the normative data of MT and RNFL thickness in Indians. They reported normal central foveal thickness of 260.1 ± 18.19 um. The nasal inner quadrant showed maximum retinal thickness (338.88 ± 18.17 um). The mean RNFL thickness was 101.43 ± 8.63 um with maximum thickness in the inferior quadrant. El-Hifnawy et al. [24, 25] collected normative MT and RNFL thickness data in the Egyptian population, the mean CST was 262.70 ± 19.64 μm and the mean NFLT was 101.74 ± 10.05 μm. The mean MT values were significantly greater in men than in women and were found to be less than those seen in the studies published previously on Caucasians using Spectralis SD-OCT.

Although the values in our study are in the normative range of other studies, SD-OCT measurements cannot be compared between different machines because each technology’s normative database is derived from a different population of different racial or ethnic groups. Sex and age also have an influential effect.

## Conclusions

We conclude that women who are using OCP for more than 1 year could develop some eye problems which could involve the central vision. We also think that using OCP with different preparations could give different results. Further long term studies are required using different preparations of combined oral contraceptive pills (COC) or progestin-only contraceptive pills (POC) to find out what are the consequences of these changes when these thickness alterations can be clinically significant or symptomatic and if these changes are reversible or not. OCT should be routinely done for follow up these cases. According to these findings, physicians should take into consideration patients ocular history, before selecting the method of contraception and before prescribing OCP for reasons other than contraception.

## Data Availability

The data used to support the findings of this study are included in the article.

## Abbreviations

BMI: Body Mass Index
COC: Combined Oral Contraceptive Pills
CSF: Central Subfield
CST: Central Subfield Thickness
CT: Choroidal Thickness
GCL: Ganglion Cell Layer
MT: Macular Thickness
MV: Macular Volume
OCP: Oral Contraceptive Pills
POC: Progestin-Only Contraceptive Pills
RNFL: Retinal Nerve Fiber Layer
SD-OCT: Spectral Domain Ocular Coherence Tomography
SD-OCT: Spectral- Domain Ocular Coherence Tomography
